# Detecting Single-Nucleotides by Tunneling Current Measurements at Sub-MHz Temporal Resolution

**DOI:** 10.3390/s17040885

**Published:** 2017-04-18

**Authors:** Takanori Morikawa, Kazumichi Yokota, Sachie Tanimoto, Makusu Tsutsui, Masateru Taniguchi

**Affiliations:** The Institute of Scientific and Industrial Research, Osaka University, 8‐1 Mihogaoka, Ibaraki 567-0047, Japan; takanori.morikawa32@sanken.osaka-u.ac.jp (T.M.); yokota@sanken.osaka-u.ac.jp (K.Y.); sachie.tanimoto32@sanken.osaka-u.ac.jp (S.T.); taniguti@sanken.osaka-u.ac.jp (M.T.)

**Keywords:** tunneling current, single molecule, sequencing

## Abstract

Label-free detection of single-nucleotides was performed by fast tunneling current measurements in a polar solvent at 1 MHz sampling rate using SiO_2_-protected Au nanoprobes. Short current spikes were observed, suggestive of trapping/detrapping of individual nucleotides between the nanoelectrodes. The fall and rise features of the electrical signatures indicated signal retardation by capacitance effects with a time constant of about 10 microseconds. The high temporal resolution revealed current fluctuations, reflecting the molecular conformation degrees of freedom in the electrode gap. The method presented in this work may enable direct characterizations of dynamic changes in single-molecule conformations in an electrode gap in liquid.

## 1. Introduction

Tunneling current measurements in liquid have been considered a promising way to identify the sequence of base molecules in RNA and DNA [1,2,3]. The method uses a pair of metal electrodes, mostly made of Au, with nanometer separation to detect temporal changes in the two-probe dc current at several to 10 kHz sampling rates upon trapping/detrapping of individual nucleobases in the nanogap [4,5,6,7,8]. Single-molecule signals were then obtained in forms of current spikes, whose height represents the electron transport through a molecule. Although the signal height and width vary widely due to the sensitive nature of the tunneling conductance on the molecular conformations, it was demonstrated that the types of nucleotides can be discriminated by analyzing and comparing the statistical distributions of the current spike height [4,5,6,7,8,9,10].

**Figure 1:**
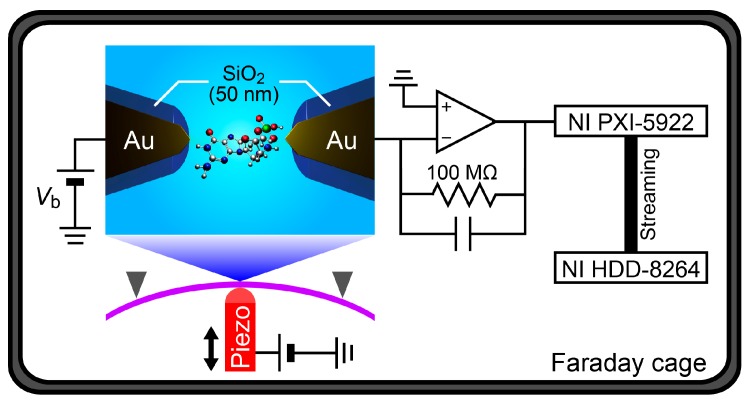
Schematic description of the tunneling current measurement setup. SiO_2_-coated Au nanoelectrodes were connected to feedthrough for biasing the dc voltage *V*_b_ and amplifying the current by the amplifier. The output voltage was digitized and stored in a hard disk drive.

Tremendous efforts have been undertaken to realize applications of the tunneling current approach for practical uses by incorporating nanopore technologies to the quantum mechanical approach, which enables the active drawing of single-polynucleotides in the gap by means of an electrophoretic control of the translocation dynamics [11,12,13,14,15]. In contrast to the progress, however, little efforts have been made to evaluate and enhance the temporal resolution of the tunneling current measurements, which is an important issue in respect to the nanopore sequencing wherein the constituent nucleobase molecules in DNA move through the electrode gap swiftly within microseconds [12,13]. In this study, therefore, we developed a nanoelectrode system for single-nucleotide detections by tunneling current measurements at 1 MHz sampling rates in liquid (Figure 1). We used insulator-protected mechanically-controllable break junctions (MCBJs) [16,17,18] to form nanotip-exposed Au electrodes of 1 nm separation. The feasibility of the nanoprobes was evaluated by carrying out single-nucleotide detections in a polar solvent at room temperature on the basis of fast tunneling current measurements at 1 MHz. 

## 2. Materials and Methods 

**Figure 2:**
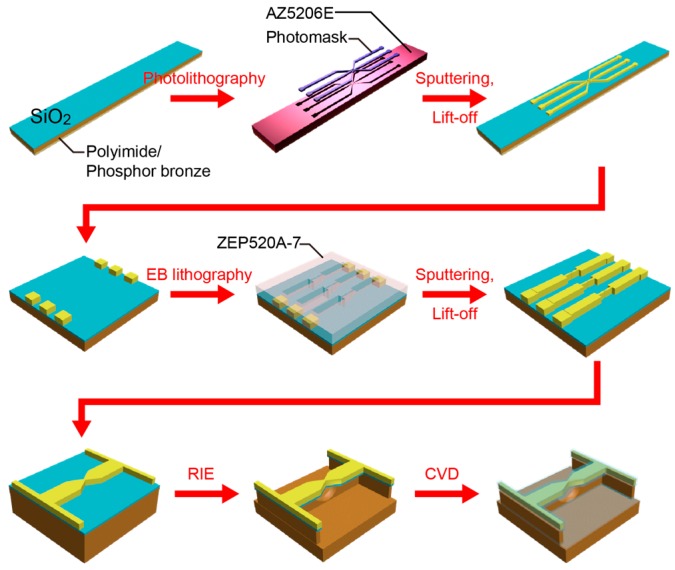
Fabrication processes of SiO_2_-coated mechanically-controllable break junctions (MCBJs).

SiO_2_-coated MCBJs were fabricated by the following processes (Figure 2). First, a polyimide layer was formed on a phosphor bronze substrate by spin coating an imide precursor (Aldrich Co., London, UK), followed by baking for polymerization. On the polymer surface, a microelectrode pattern was delineated by a photolithography method using AZ-5206E resist (Zeon Co., Tokyo, Japan). After development, a 40-nm thick Au layer with a 5-nm thick Cr adhesion layer was deposited by radio-frequency magnetron sputtering. The sample substrate was then immersed in N,N-dimethylformaide (Wako Co., Tokyo, Japan) overnight and ultrasonicated for lift-off. Further, nanojunctions were drawn by an electron-beam lithography using ZEP520A resist (Zeon Co.). Subsequently, a 100-nm thick Au layer together with a 1-nm thick Cr adhesion layer was deposited by the sputtering process. As a result, we obtained Au nanojunctions after lift-off in N,N-dimethylformamide. Finally, we dry-etched the polyimide layer by a reactive ion etching with oxygen etchant gas to partially free the junctions from the substrate. Here, the length of the free-standing Au nanobridges was designed to be approximately 2 μm, which is an optimal design for the MCBJ setup used in the present study; while shorter bridges would render better mechanical stability and finer control of the electrode gaps, they also become critically difficult to break by bending the substrate. The actual displacement rate was calibrated by analyzing the interelectrode distance dependence of the tunneling current, as described elsewhere [16,18].

In the experiments, an MCBJ sample was mounted on a stage in a three-point bending configuration. Then, a dilute solution (0.1 mM) of deoxycytidine monophosphate (dCMP) or deoxyguanosine monophosphate (dGMP) was poured into a Teflon cell attached to the MCBJ. The phosphor bronze substrate was then deflected by bending it using a piezo-driven pushing rod. Meanwhile, the conductance *G* of the junction was measured under the applied dc bias voltage *V*_b_ using a picoammeter/source unit (Keithley 6487). By bending the substrate, *G* tended to decrease gradually due to necking deformation at the narrowest constriction of the junction via tensile force. Further bending led the junction to break, as denoted by the *G* drop to zero. The thus-created electrode gap was adjusted to be 1 nm by the piezo-control (details are described in [18]).

After forming the nanoelectrodes, the measurement unit was switched from the picoammeter to a custom-built current amplifier based on an operational amplifier ADA4817 and a 100 MΩ feedback resistance with a gain and bandwidth of 10^8^ V/A and >1 MHz [19], respectively. The voltage source was also changed to a battery for the sake of attaining low noise. The two-probe current was then measured at *V*_b_ = 0.5 V by recording the amplifier output using a digitizer (NI PXI-5922) and a RAID system (NI HDD-8264) at a 1 MHz sampling rate. More than three MCBJ devices were used to correct the tunneling current spike data for each nucleotide measured.

## 3. Results

**Figure 3:**
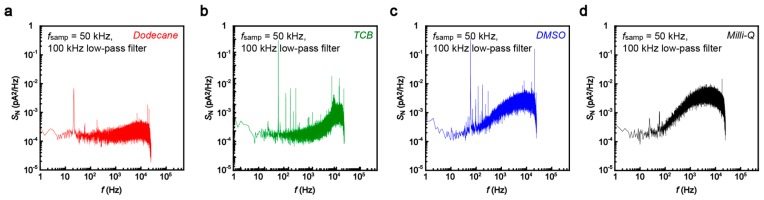
(**a**–**d**)**,** Noise spectra obtained from the current versus time curves recorded at 50 kHz with a 100 kHz low-pass filter using an Au electrode gap of a size larger than 1 nm in dodecane (**a**); 1,2,4-trichrolobenzene (**b**); dimethyl sulfoxide (**c**); and Milli-Q (**d**).

**Figure 4:**
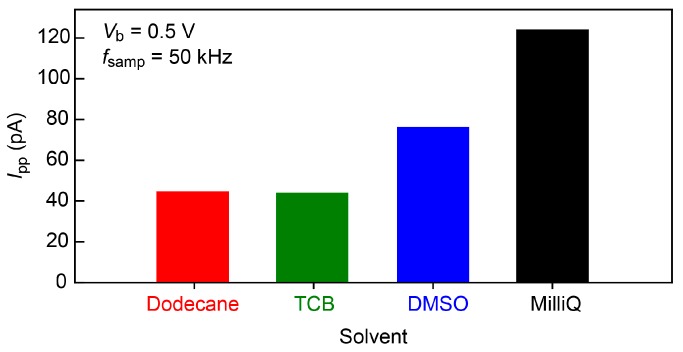
Solvent dependence of current noise measured with 1 nm-sized SiO_2_-protected Au electrode gaps. Sampling rate was 50 kHz. 100 kHz low pass filter was used for the current measurements. The noise is described as peak-to-peak values obtained in dimethyl sulfoxide (DMSO), dodecane, 1,2,4-trichrolobenzene (TCB), and Milli-Q.

Fast current measurements generally involve increased noise. On the other hand, the single-nucleotide conductance only provides current changes as much as sub nanoamperes at *V*_b_ = 0.5 V [4,5]. We therefore tested several organic solvents, dodecane, 1,2,4-trichlorobenzene (TCB), and dimethyl sulfoxide (DMSO), in addition to water in order to obtain a low-noise condition for the single-molecule detections. The noise was characterized in terms of the power spectrum density *S*_N_ calculated from the current versus time curves recorded in the solvent at 50 kHz sampling rates with 100 kHz low-pass filtering (Figure 3). The noise spectra showed linear components at the frequency *f* above 10^2^ Hz. This feature is naturally interpreted as stemming from the voltage noise in the current amplifier coupled to the net capacitance of the MCBJ system [19,20]. It is noticeable that the slope is steeper in DMSO and Milli-Q compared to that in dodecane and TCB. As a result, we obtained the lowest peak-to-peak noise *I*_p-p_ in dodecane and TCB (Figure 4). The noise increased by a factor of 1.7 and 2.8 in a polar organic solvent, dimethylsulphoxide (DMSO), and water, respectively. This suggests the importance of the role of the capacitance of electric double layers (EDLs) formed on the biased electrode surface that serve to increase *I*_p-p_ through interaction with the noise in the amplifier voltage [19,20]; while the polar DMSO as well as water molecules accumulate on the electrode surface to screen the electric field there, dodecane is non-polar and hence does not form dense EDLs. More specifically, relative polarity indices *P*_x_ of the solvents are *P*_water_ > *P*_DMSO_ > *P*_dodecane_ ~ *P*_TCB_ [21], which yields the EDL capacitance, and hence *I*_p-p_, of the same order. In this respect, dodecane or TCB was found to be the best choice for the single-molecule detections. Unfortunately, however, nucleotides cannot be dissolved in the non-polar solvent. We therefore employed DMSO and Milli-Q to perform the single-molecule tunneling current measurements.

**Figure 5:**
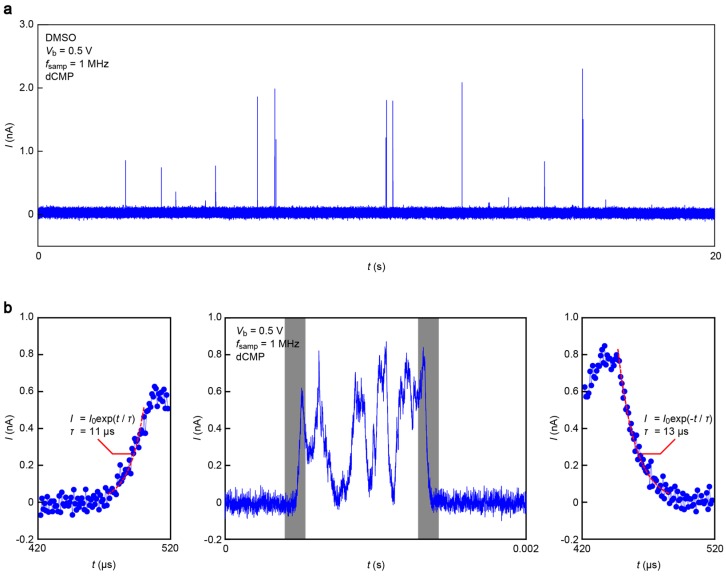
(**a**) Current versus time trace recorded in a DMSO solution of dCMP using a 1 nm-sized SiO_2_-protected Au electrode gap at a 1 MHz sampling rate with a 1 MHz low-pass filter under the applied voltage *V*_b_ of 0.5 V. Pulse-like signals were observed, signifying trapping/detrapping of single-molecule dCMP between the nanoprobes; (**b**) Magnified views of a tunneling current spike. Grey regions in the middle panel indicates the rising (left) and falling (right) of the signal. Red dashed lines represent the exponential fit.

Figure 5 displays a current (*I*) versus time (*t*) trace obtained in a DMSO solution of monomer dCMP at 1 MHz sampling rate and *V*_b_ = 0.5 V. We observed *I* spikes suggestive of a transient increase in the tunneling current upon single-nucleotide trapping/detrapping between an electrode gap (Figure 5a). A close look at each pulse signal revealed fast current fluctuations attributable to dynamic changes of the molecular conformations in a stochastic manner (Figure 5b, middle) [13]. It is also worth noting that the *I*-*t* feature is somewhat blunt. 

**Figure 6:**
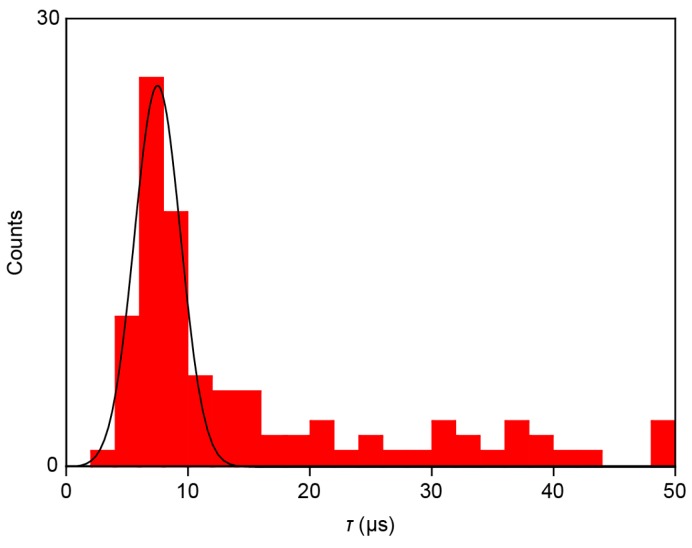
Statistical distribution of the time constant *τ*. Solid curve is a Gaussian fit.

The smooth changes in *I* can be attributed to the slow dynamical motions of dCMPs being captured and escaping from the electrode gaps. However, the molecular motion-derived tunneling current fluctuations were predicted to take place at a pico-second time-scale [13]. The microsecond response of *I* is, therefore, an unlikely intrinsic characteristic of tunneling transport through Au-dCMP-Au systems. On the other hand, previous studies reported significant *RC* contributions to retard the rapid change of the tunneling current detected in aqueous media due in part to large capacitance at the EDLs formed on the current sensing electrode surface, as well as that of the insulator layer [19]. Indeed, we found exponential changes in *I* in the rise and fall traces (Figure 5b, left and right panels), suggesting the influence of the capacitance effects. For these curves, numerical fitting gave the time constant *τ* of 11 μs and 13 μs, respectively, during the single-molecule trapping and detrapping. Furthermore, a statistical analysis of *τ* calculated for 78 spikes measured showed a monomodal distribution centered at 7.5 μs (Figure 6). This indicates the existence of resistance and capacitance components amounting *RC* = 7.5 μs in the measurement circuit, including the MCBJ device used. Meanwhile, the non-Gaussian distribution at the high-*τ* regime would be attributed to non-specific adsorption of nucleotides on the Au surface virtually slowing down the detrap dynamics.

**Figure 7:**
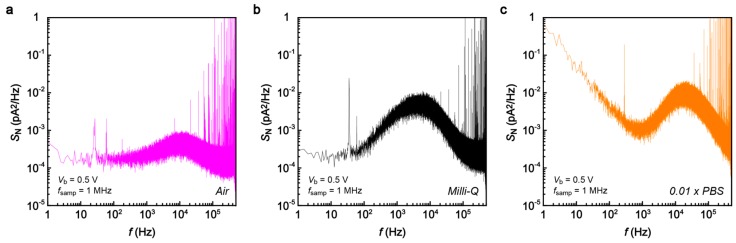
(**a**–**c**) Noise spectra in an air gap (**a**); Milli-Q (**b**); and 0.01 × PBS (**c**) at 1 MHz sampling rate with a 1 MHz low-pass filter.

**Figure 8:**
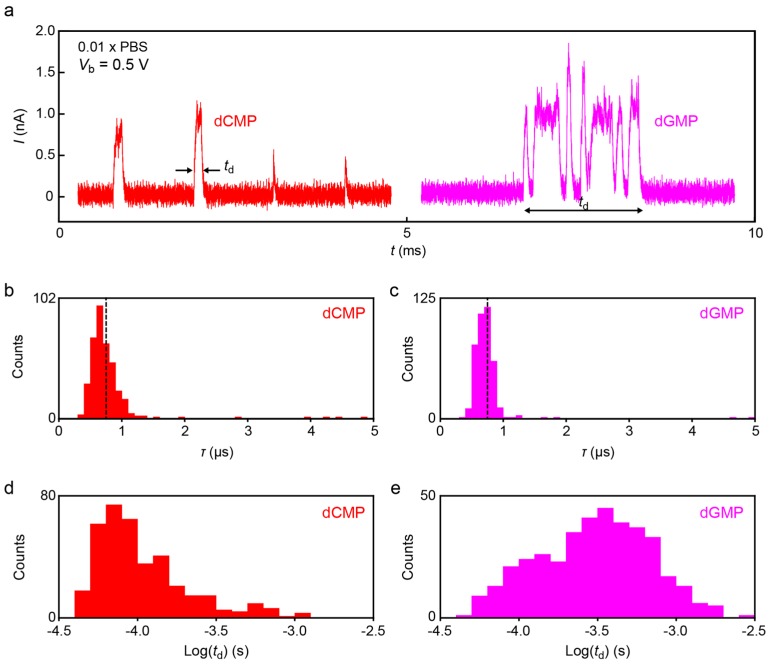
(**a**) Tunneling current spikes detected for dCMP (red) and dGMP (purple) in 0.01 × PBS at 1 MHz with a 1 MHz low-pass filter; (**b**,**c**) Distributions of *τ* for dCMP (**b**) and dGMP (**c**) in 0.01 × PBS. Dotted lines denote the average *τ* in DMSO; (**d**,**e**) Spike width *t*_d_ histograms for dCMP (**d**) and dGMP (**e**).

To shed further light on this point as well as to testify the feasibility of the 1 MHz current sampling in more practical conditions, we extended the tunneling current measurements in diluted PBS buffer (×0.01) for detections of dCMP and dGMP. In contrast to the noise characteristics in the air gap (Figure 7a) demonstrating mostly Johnson noise contributions, we observed pronounced capacitance effects (*S*_N_ ~ *f*) in Milli-Q as well as in the buffer solution at *f* > 10^2^ Hz, as expected from the high ion concentration condition that leads to formation of dense EDLs on the electrode surface (Figure 7c). Moreover, ions in the salt solution give pronounced flicker noise that further deteriorates the noise condition, giving *I*_pp_ of 447 pA at *V*_b_ = 0.5 V. Fortunately, however, the noise level was not critical for detecting the tunneling current signatures of nucleotides. Figure 8 displays typical spike signals obtained for dCMP and dGMP (Figure 8a) in the diluted buffer demonstrating large fluctuations of the tunneling current attributable to dynamic changes in the molecular conformations in the electrode gap. *τ* deduced from the spike tails revealed monomodal distributions positioned at *τ* = 6.7 μs and 6.9 μs for dCMP and dGMP, respectively (Figure 8b,c), which is quite close to that in DMSO. The fact that *τ* changes little between the two nucleotides indicates the predominant influence of signal retardation by the *RC* effects on *τ*.

While no notable molecular feature was found in the *τ* distributions, we observed clear difference in the spike width *t*_d_. Figure 8d,e shows *t*_d_ histograms for dCMP and dGMP. The results reveal longer *t*_d_ of dGMP than dCMP, indicating that the former molecule tends to be trapped for a longer period in the electrode gap. This difference could not be observed conspicuously in the previous experiments due in part to the inadequate sampling rate (several kHz) to correctly detect the short-lived trapping events with *t*_d_ in a range of sub-milliseconds. We hope that future works clarify the underlying mechanism of the nucleotide-dependent trap durations.

## 4. Discussion

**Figure 9:**
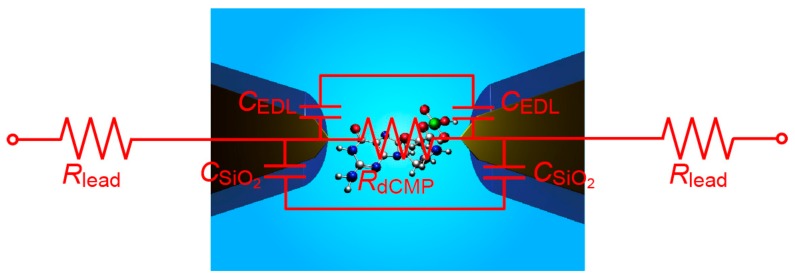
Rough circuit model of the SiO_2_/Au nanoelectrodes consisting of the single-molecule resistance (*R*_dCMP_), the capacitance of the electric double layers (*C*_EDL_) and SiO_2_ layers (*C*_SiO2_), and the Au lead resistance (*R*_lead_).

It is interesting to consider the source of *RC* element relevant to the temporal resolution of the tunneling current measurements in the present setup. For the SiO_2_-covered Au nanoelectrodes, the structure can be roughly described by an equivalent circuit consisting of the resistance *R*_dCMP_ of single-molecule dCMP and the capacitance of EDL, *C*_EDL_, and the SiO_2_ layer, *C*_SiO2_, connected in parallel, together with the resistance of Au lead, *R*_lead_, connected in series (Figure 9). Here, *R*_dCMP_ = 35 GΩ on average [4], *C*_SiO2_ = *Wεε*_0_/*L* = 7 pF considering the surface area of SiO_2_/Au micro-leads of width *W* and length *L* of 10 μm and 1 mm, respectively (with the vacuum permittivity *ε*_0_ and the relative permittivity of SiO_2_ *ε* = 3.9), and *C*_EDL_ < 1 fF at the exposed Au tip surface with an of area 100 nm × 100 nm. These values give *RC*_gap_ at the electrode gap of about 0.1 s, where *C*_gap_ = (*C*_SiO2_/2 + *C*_EDL_/2) is the capacitance of the electrode gap, which is considerably longer than what is found in the tunneling current response in the present study. On the other hand, Smeets et al. [22] proposed empirically that the bandwidth of the ionic current measurements in a nanopore system is determined by the product of the membrane capacitance and the access resistance, instead of the resistance inside the pore [22,23]. Analogously, although the underlying physical mechanism is still elusive, *τ* in the present work may also be determined by *R*_lead_ rather than *R*_dCMP_. In that case, tentatively assumed *R*_lead_ = 1/G_0_ = 12.9 kΩ provides *R*_lead_*C*_EDL_/2 = 7 μs with *C*_EDL_ = 1 nF for the non-protected MCBJs, which is in agreement with the experimental *τ* of 45 μs within an order of magnitude. Meanwhile, the reduced *C*_EDL_ in the SiO_2_-coated nanoelectrodes predicts *R*_lead_*C*_gap_ ~ *R*_lead_*C*_SiO2_/2 of 90 ns. This indicates that the temporal resolution is limited by a parasitic capacitance much larger than *C*_SiO2_. Further effort to reduce the capacitance in the measurement system, for instance via analog compensation [23], may improve the temporal resolution beyond MHz.

## 5. Conclusions

We performed single-nucleotide detections by fast tunneling current measurements at 1 MHz sampling rates using SiO_2_-protected nanoelectrodes. We observed rapid fluctuations of the current within individual spike signals, signifying dynamic molecular conformation changes in the electrode gap. Meanwhile, blunt features were found in the single-molecule traces, which indicated signal retardation by *RC* effects limiting the temporal resolution to around 7 μs. Further efforts to reduce the capacitance may enable fine tracking of tunneling current changes in an aqueous solution for studying single-molecule dynamics at high spatiotemporal resolutions, thus delivering one of the essential technologies to realize sequencing by quantum mechanics.
